# Analysis of the non-reciprocating legged gait for a hexapod robot based on the ePaddle-EGM

**DOI:** 10.1186/s40638-016-0034-2

**Published:** 2016-06-28

**Authors:** Jinglei Zhao, Huayan Pu, Jun Zou, Yi Sun, Shugen Ma

**Affiliations:** School of Mechatronic Engineering and Automation, Shanghai University, 149 Yanchang Road, Shanghai, China; Engineering Training Centers, Guizhou University, Guiyang, Guizhou China; Department of Robotics, Ritsumeikan University, Shiga, 525-8577 Japan

**Keywords:** ePaddle-EGM, Non-reciprocating kinematic method, Hexapod robot

## Abstract

A novel eccentric paddle mechanism based on the epicyclic gear mechanism (ePaddle-EGM) has been proposed to enhance the mobility of amphibious robot for multi-terrain tasks with diverse locomotion gaits. This paper presents a brief description for this mechanism. Based on the feature of ePaddle-EGM, a unique non-reciprocating legged gait planning method is proposed. This method could minimize the negative effect of backlash between gear mesh in the epicyclic gear mechanism. Furthermore, the stable tripod gait for the ePaddle-EGM-based hexapod robot is designed. One of the most important characteristics of this tripod gait is that it is capable of realizing discontinuous locomotion of the body through continuous and unidirectional rotation of joints. In this way, the velocity shock is eliminated and the locomotion accuracy is guaranteed. A series of simulations were conducted to validate the advantages of the robot’s movement.

## Background

Due to the strong environmental adaptability in harsh environments such as land, water and beach, amphibious robots can perform various tasks for human, such as environmental monitoring, resource exploration, disaster rescue and underwater mine clearing. This makes amphibious robots have a wide application prospect. Researchers have paid more and more attentions to the novel structure design, control and navigation of the amphibious robot, to improve its mobility in multi-environments. Various types of amphibious robots have been built. One of the most remarkable types is the biomimetic amphibious robot, which is inspired from the morphological feature of natural creatures. By learning and imitating the biologic characteristics and function of their biologic counterparts, this type of amphibious robots then reproduces these characteristics, such as Salamander I [1] and II [2] by Crespi, Pleurobot by Karakasiliotis [3], ACM-R5 by Yamada [4], Ariel by Yamauchi [5] and MLMR II by Deng [6]. Besides, researchers have also developed several novel robotic mechanisms to achieve amphibious mobility, by integrating several basic motion units into one locomotion mechanism. For instance, the wheel–leg–tail integration robot DAGSI Whegs developed by Boxerbaum [7], paddle–wheel integration autonomous amphibious vehicle developed by Frejek [8] and leg–paddle integration robot AQUA [9].

Instead of focusing on the design of propulsive mechanism for simple environment, we have proposed a novel hybrid-mechanism-based amphibious propulsive mechanism in our previous works [10, 11], and it is called the ePaddle-EGM. It is made up mainly of an eccentric paddle and an epicyclic gear train. The ePaddle-EGM is an innovative mechanism which combines wheeled, legged and paddling gaits to perform multi-terrain locomotion for amphibious tasks [12, 13]. High motion performance on multiple terrains can be simply achieved by actively adjusting the position of paddle shaft in the ePaddle mechanism.

However, some problems about the ePaddle-EGM were exposed as well. For example, the backlash between gear meshes may compromise the accuracy of locomotion in legged walking or aquatic paddling modes. Therefore, in order to promote motion accuracy with the natural presence of backlash, we proposed a novel kinematic method based on the idea of generating reciprocating trajectory of the paddle using unidirectional and continuous rotations of the actuators.

In this paper, we briefly introduce the non-reciprocating kinematic method which can overcome the negative influence of backlash. The rest of this paper is organized as follows. The introduction of the ePaddle-EGM module, the non-reciprocating kinematic method and the gait planning method of the ePaddle-based hexapod robot are presented in section ‘Methods.’ The results of simulations for ePaddle-based hexapod robot are listed in section ‘Results and discussion.’ Finally, section ‘Conclusions’ concludes this paper.

## Methods

### The eccentric paddle mechanism

**Fig. 1:**
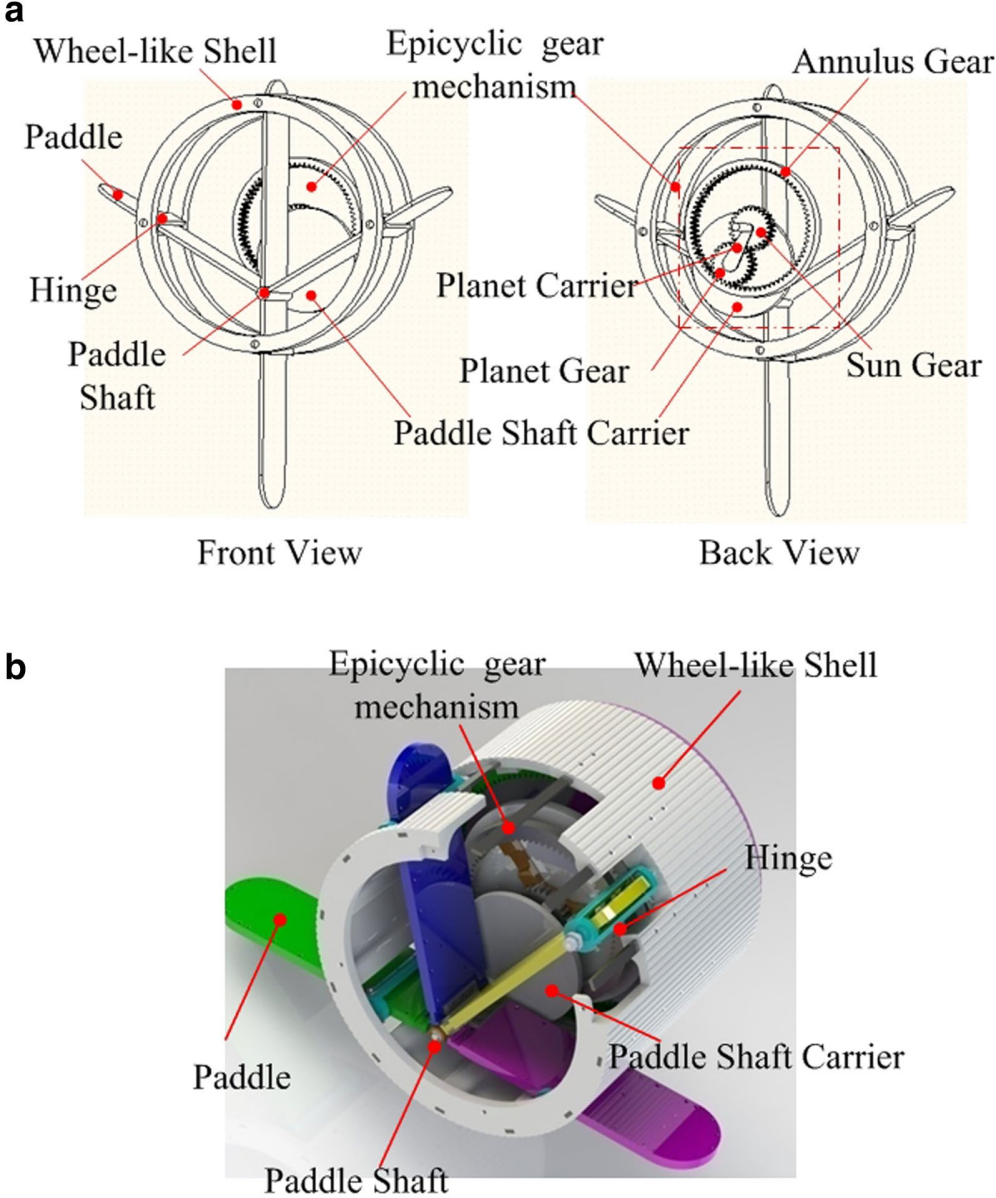
Structure sketch of the ePaddle-EGM. **a** Structure sketch. **b** CAD model

The eccentric paddle mechanism is based on epicyclic gear mechanism, which can perform several locomotion gaits. Figure 1 shows the concept and prototype of the ePaddle-EGM module, respectively. One ePaddle-EGM module is composed of four main components: (1) a movable paddle shaft that is driven by an epicyclic gear mechanism, (2) a set of four paddles that can rotate around that paddle shaft freely, (3) an actively actuated shell that can rotate around the fixed shell axis and (4) four passive paddle hinges that are located radially at the edge of the shell and allow the paddle to slide through them. When the shell is driven to rotate around the shell shaft, hinges force the paddles to rotate around the paddle shaft accordingly. Because the paddle shaft is actively located inside of the shell by an epicyclic gear mechanism, the motion pattern of the tip point of the paddles can be alternated.

### Non-reciprocating kinematic method

The application of epicyclic gear mechanism has brought plenty of advantages for our design, such as the high reliability and high efficiency of the gear train. But at the meantime, the backlash between gear mesh may accumulate during locomotion and then influence the accuracy of the locomotion seriously. On the other hand, generally, the active joints of multi-legged robot have to change their direction of motion frequently during the successive locomotion of robot. The change in the direction of motion may bring in sudden change in joint velocity. So based on these consideration, we have proposed the non-reciprocating kinematic method which will be presented below.

#### Related definitions

Some of the technical terms needed to describe the gait should be defined before we can proceed further [14].

A stride is a complete cycle of leg movements, for example, from the setting down of a particular foot to the next setting down of the same foot.

A gait cycle *T* is the duration of a stride. For legged robot, the gait cycle consists of the stance phase (STP) and the swing phase (SWP). The duty factor *ξ* is the ratio of duration of STP to a whole gait cycle; in other words, *ξ* of a foot is the fraction of the duration of the stride for which it is on the ground. In nearly all the gaits used by animals, the left and right feet of a pair have approximately equal duty factors.

**Fig. 2:**
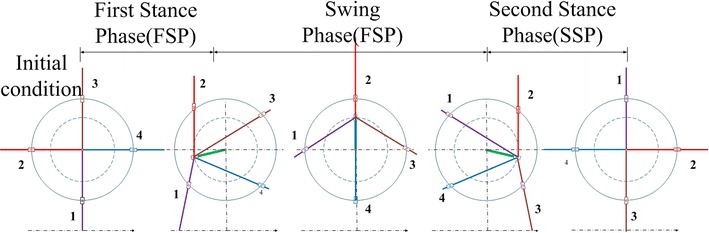
Locomotion of ePaddle-EGM in one cycle

Furthermore, in this paper, according to the proposed method, we divided the stance phase into two fractions, namely the first stance phase (FSP) and the second stance phase (SSP), as shown in Fig. 2. We also define the ratio of duration of FSP to STP as stance factor *j*.

*λ*_FSP_/*λ*_SSP_ means the distance of end of each leg traveled in the horizontal direction relative to the hip joint in the first/second stance phase, and *λ* is the sum of *λ*_FSP_ and *λ*_SSP_; detailed information could be found in article [15, 16].

#### Features of proposed method

For purpose of improving the motion accuracy and efficiency, we proposed the non-reciprocating kinematic method. Since the detailed information about this kinematic method is not the focus of this paper, so it will not be described here.

The most notable feature of this method is that during the whole locomotion cycle, all the active joints in the mechanism are rotating unidirectionally and continuously. The unidirectivity of active joints can diminish the negative effect of backlash between gear mesh. Because of the continuity, the velocity shock caused by sudden change in joint velocity is also eliminated. Furthermore, since the *λ*_FST_ and *λ*_SSP_ could be arbitrary value, when the duration of first/second stance phase is constant, we can change velocity of ePaddle-EGM immediately. At present, since the rotary of each active joint is unidirectional and continuous, it means the ePaddle-EGM is capable of altering the locomotion pattern smoothly and instantaneously.

#### Examples

**Fig. 3:**
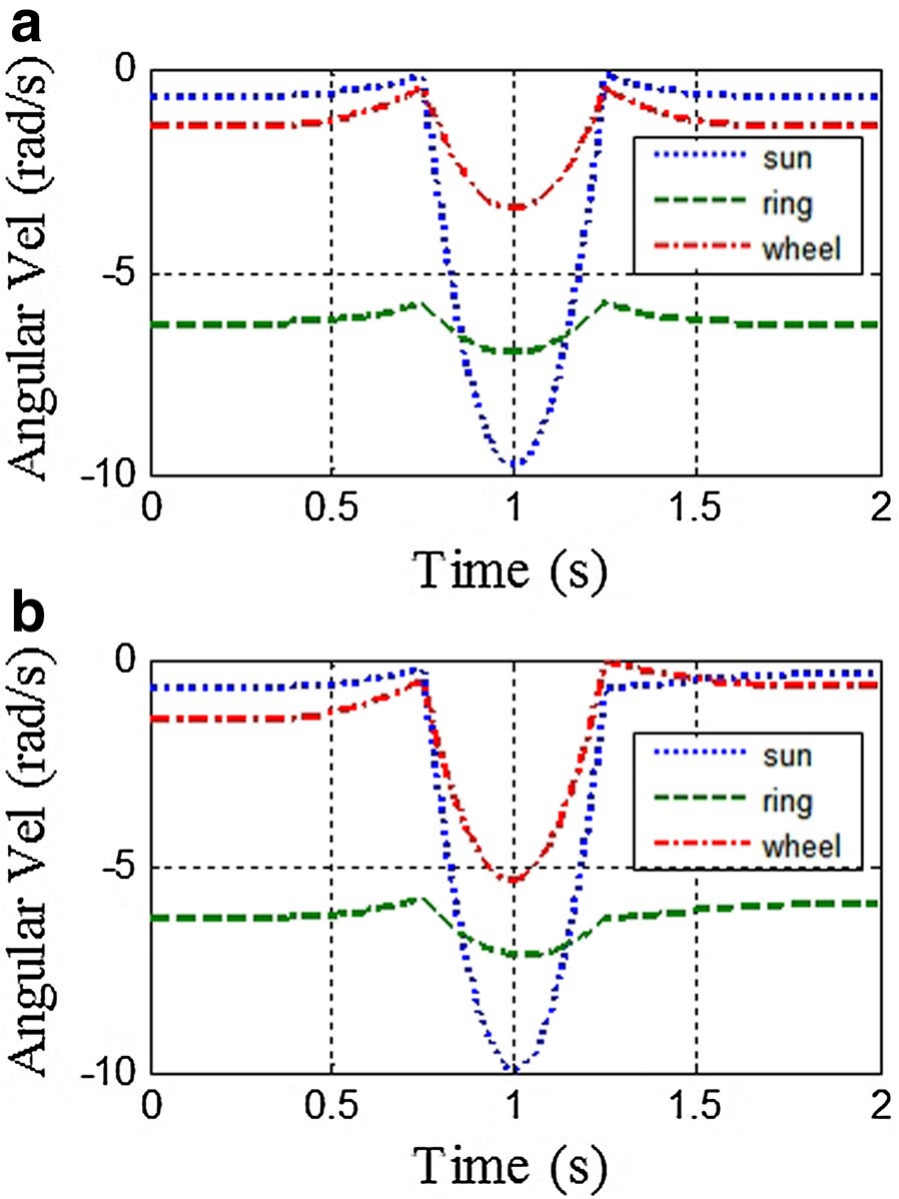
Angular velocities of actuators in the ePaddle-EGM under different initial conditions. **a** *λ* = 320 mm, *T* = 2 s, *ξ* = 0.75, *j* = 0.5. **b** *λ* = 165 mm, *T* = 2 s, *ξ* = 0.75, *j* = 0.5. The *dotted line*, the *dash line* and the *dot-and-dash line* are for the sun gear, the ring gear and the wheel respectively

Here are two examples to demonstrate the features of this proposed method. Initial conditions for these two examples are listed below: (1) *λ* = 320 mm (*λ*_FST_ = *λ*_SST_ = 160 mm), *T* = 2 s, *ξ* = 0.75, *j* = 0.5; (2) *λ* = 165 mm (*λ*_FST_ = 160 mm, *λ*_SST_ = 5 mm), *T* = 2 s, *ξ* = 0.75, *j* = 0.5. By comparing two groups of parameters, one can find that all parameters are identical except *λ*_FST_. According to the proposed kinematic method, the angular velocity of each actuator in a whole locomotion cycle under each initial condition can be attained respectively, as shown in Fig. 3. Figure 3 shows that all the curves are always below zero and continuous, which means all the motors are rotating continuously and unidirectionally during the movement. As a result, the unidirectional motions of the motors ensure the accuracy of the locomotion.

**Fig. 4:**
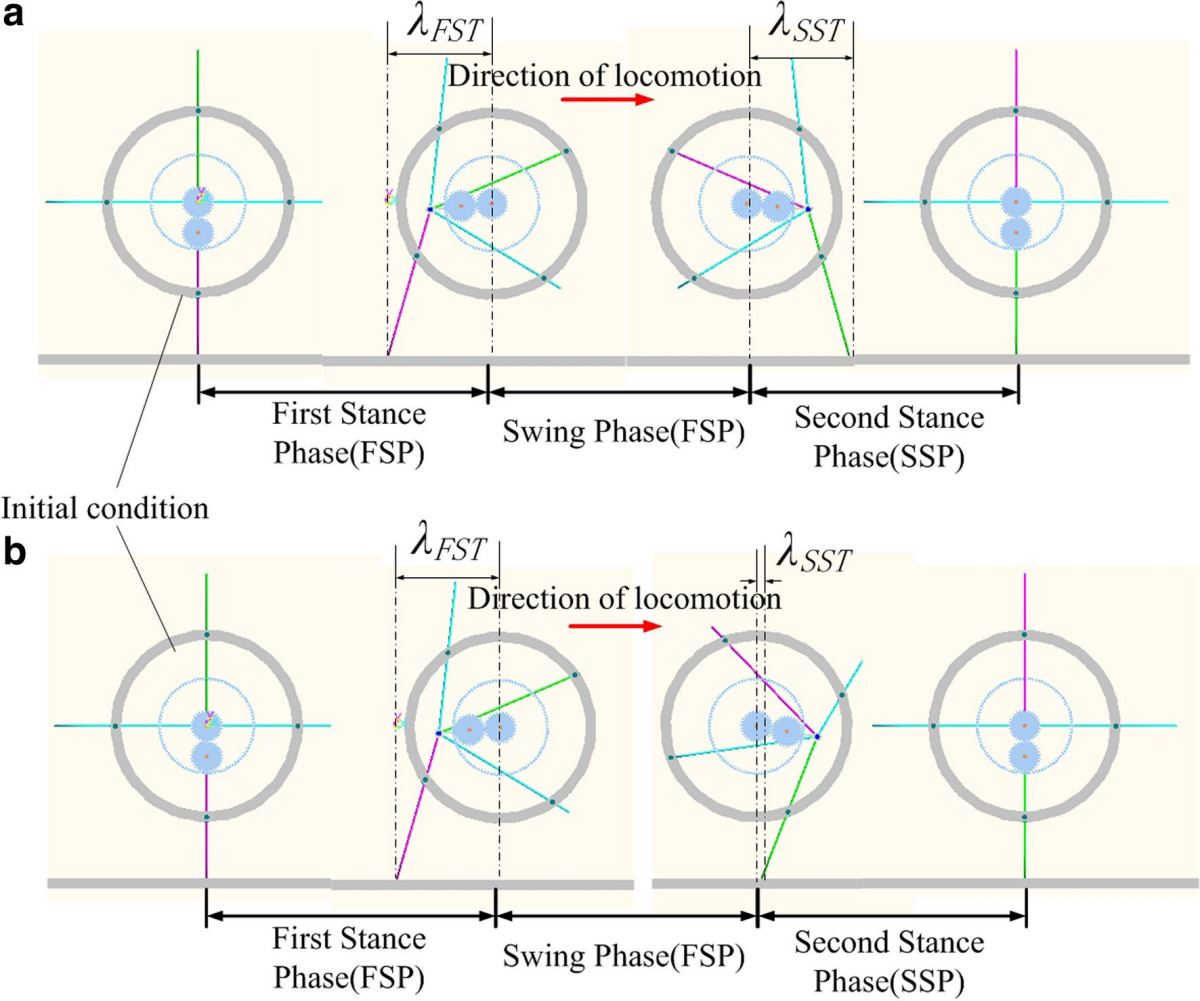
The locomotion of ePaddle-EGM relative to the world coordinate system. **a** *λ* = 320 mm, *T* = 2 s, *ξ* = 0.75, *j* = 0.5. **b** *λ* = 165 mm, *T* = 2 s, *ξ* = 0.75, *j* = 0.5

**Fig. 5:**
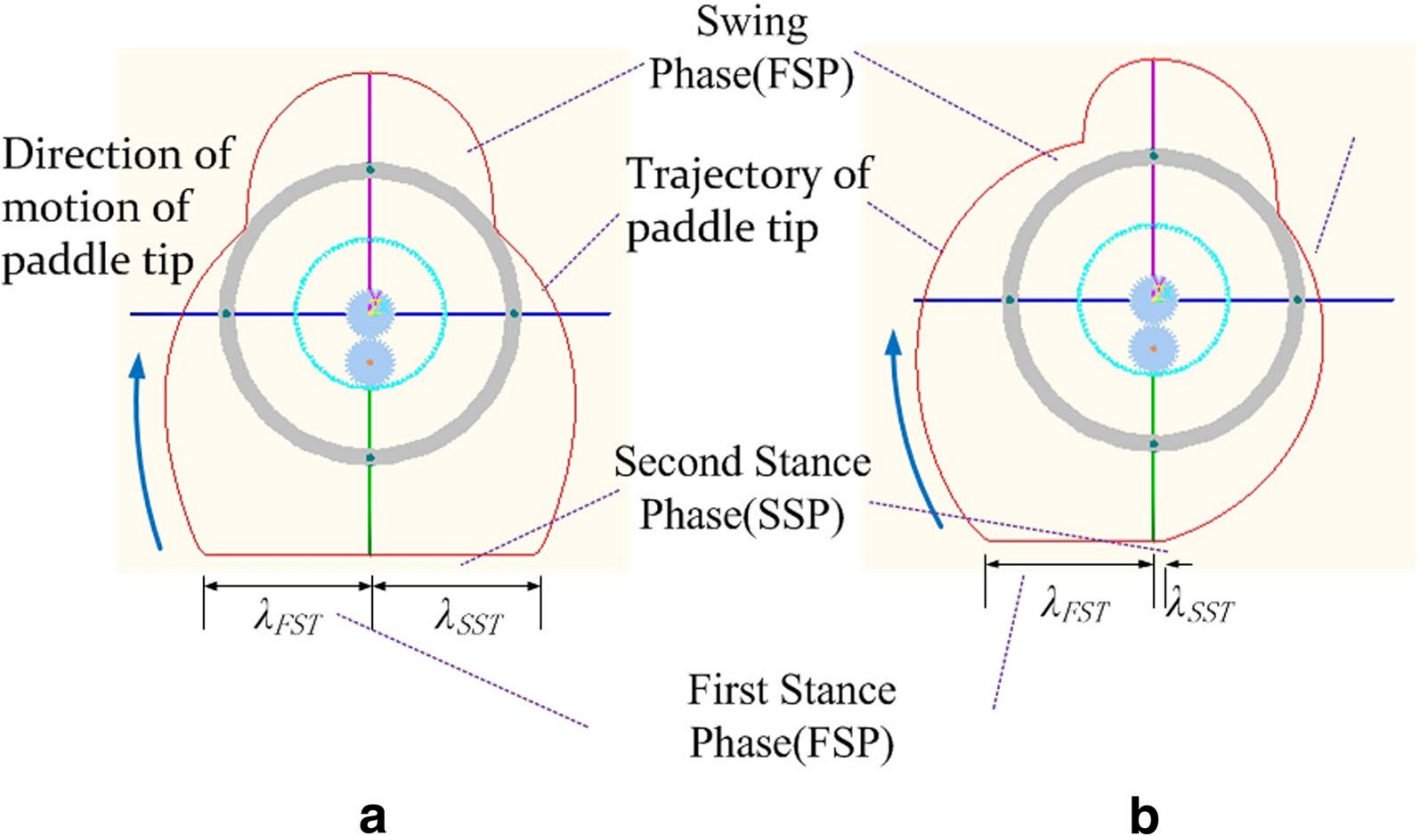
Trajectories of paddle tip relative to the body coordinate system. **a** *λ* = 320 mm, *T* = 2 s, *ξ* = 0.75, *j* = 0.5. **b** *λ* = 165 mm, *T* = 2 s, *ξ* = 0.75, j = 0.5

Movements of ePaddle-EGM and trajectories of end of the paddle under two conditions are shown in Figs. 4 and 5, respectively. It is noteworthy that *λ*_FST_ and *λ*_SST_ are not equal, so are the velocities of ePaddle-EGM in FSP and SSP. Even though the velocity of ePaddle-EGM has been changed, velocities of actuators mounted in ePaddle-EGM are still continuous and unidirectional. In other words, ePaddle-EGM has altered its motion pattern smoothly and instantaneously, i.e., has switched from one pattern which is quick to another which is much slow. This property is useful when it is applied to multi-legged robot which we will discuss next.

### The ePaddle-based hexapod robot

Generally, there are two types of gaits adopted in mobile robot—periodic gait and non-periodic gait which is called free gait as well [17]. The former could increase the adaptability of the mobile robot because it can let robot move on the uneven terrain. However, the free gait is difficult to be realized in the real multi-legged mobile robots and is only on the stage of theoretical research. Periodic gait such as tripod gait can be easily controlled and has an optimal stability margin [18]. So in this section we will be concentrated on realizing the non-reciprocating tripod gait of the ePaddle-based hexapod robot.

#### Robot design

**Fig. 6:**
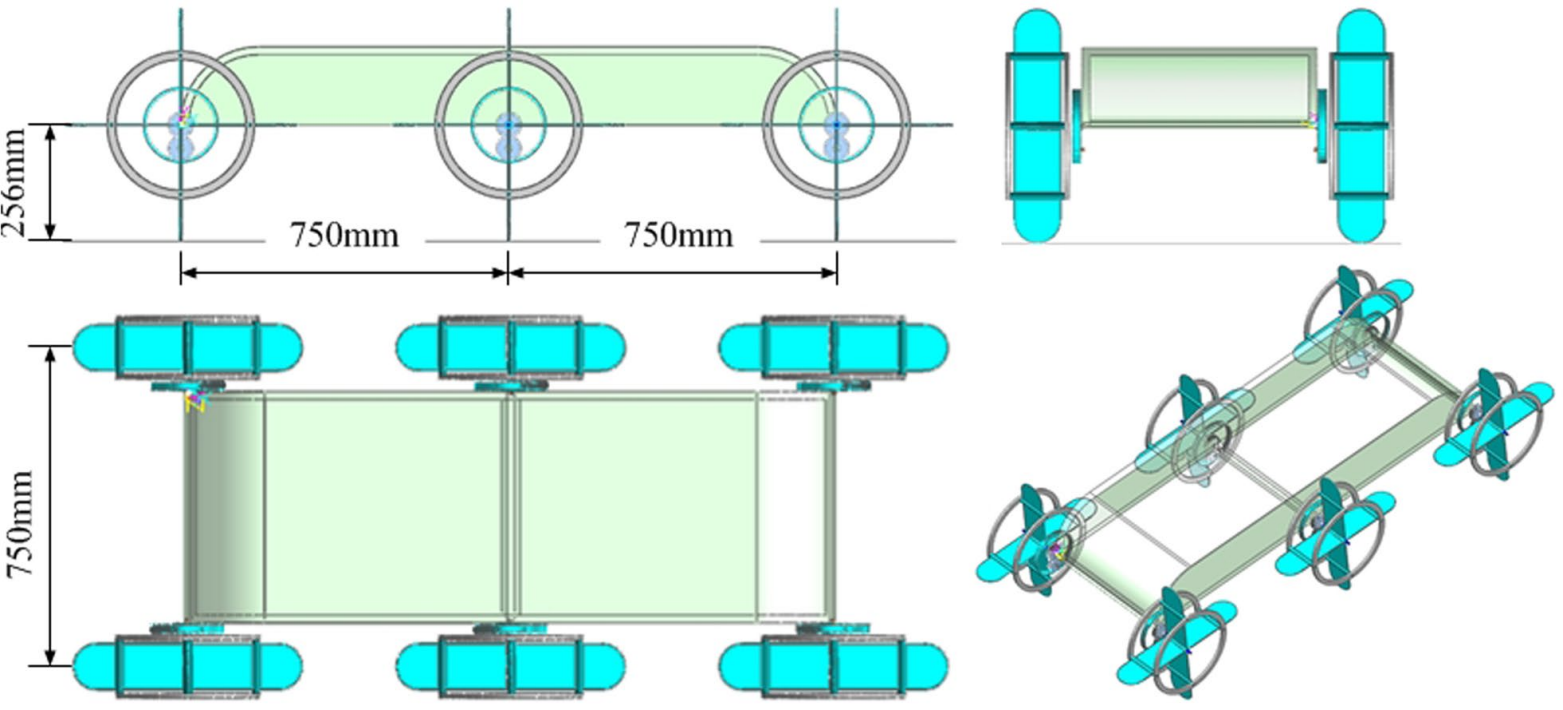
ePaddle-based hexapod robot

Figure 6 shows the model and main structural dimensions of the ePaddle-based hexapod robot that we have already developed. This hexapod robot has six identical legs (ePaddle-EGMs) aligned symmetrically along the body, being three on each side. The total length of the robot when the legs are vertical is 1.5 m, and the height of the body can change from 0 to 256 mm. Since each ePaddle-EGM has 3 DOFs, thus the robot has 18 DOFs. Next, based on this hexapod robot, we will propose the tripod gait planning method to realize the locomotion of it.

**Fig. 7:**
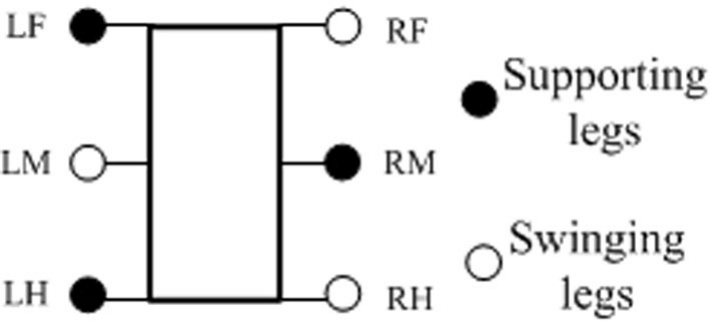
Simplified model of the ePaddle-based hexapod robot

The simplified model of robot is shown in Fig. 7. All the legs are named as left foreleg (LF), left middle leg (LM), left hind leg (LH), right foreleg (RF), right middle leg (RM) and right hind leg (RH), respectively. The six legs are divided into two sets during tripod locomotion. Each set contains the fore and hind modules of one side and middle module of the other side. Each module in the same group moves identically. Two sets of modules support the body alternately. In each ePaddle-EGM module, the duration of support phase and swing phase is the same.

#### Uniform rectilinear motion

**Fig. 8:**
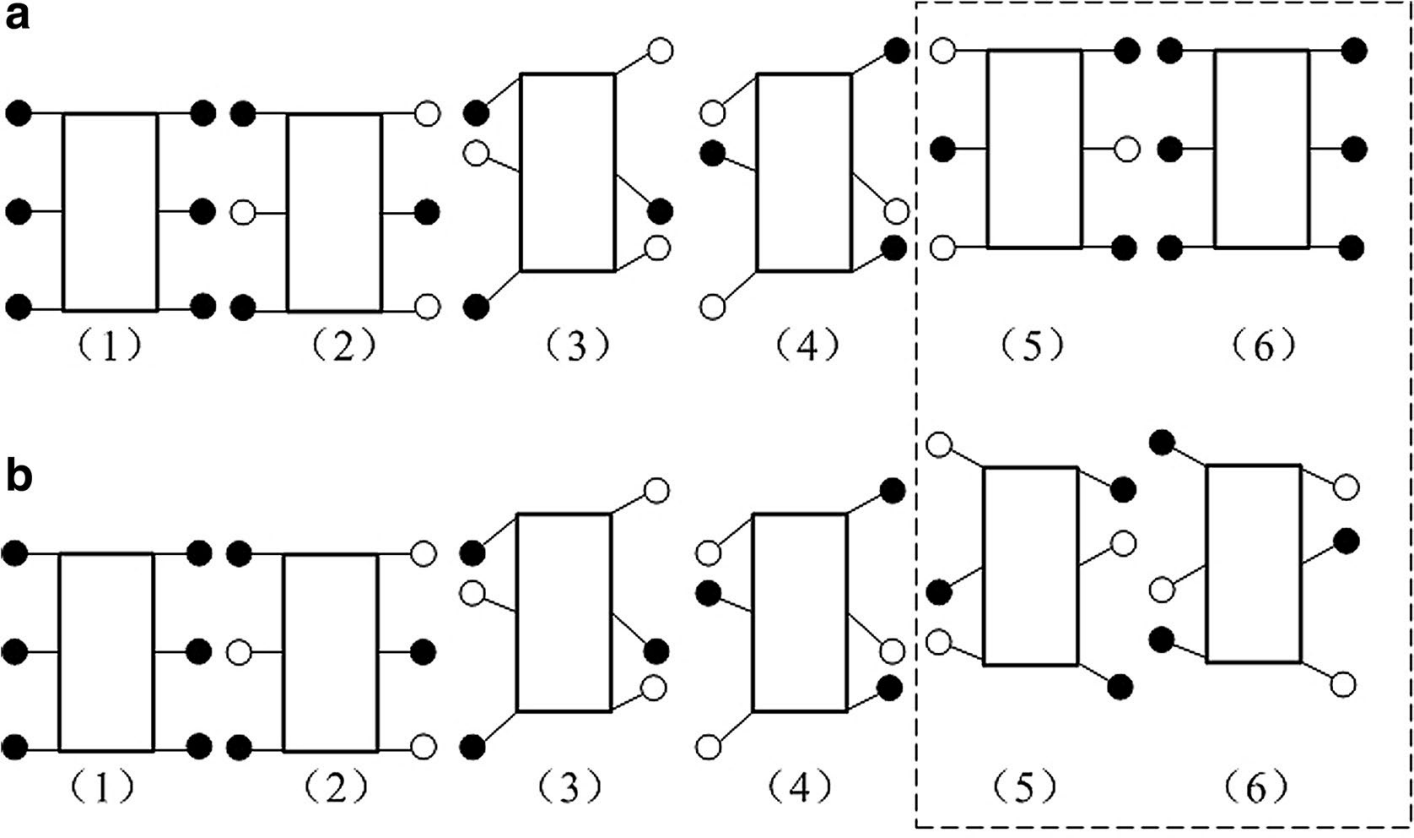
Uniform rectilinear motion. **a** Locomotion of robot only in one locomotion cycle. **b** Successive locomotion of the robot

Figure 8 shows the uniform rectilinear motion of the hexapod robot. The difference between (a) and (b) is: (a) illustrates the locomotion of robot only in one locomotion cycle and (b) illustrates the successive locomotion of the robot. By Fig. 8a, one can observe that at the beginning of the locomotion, all six legs are in FSP. Then one set of legs starts to swing, and the another set of legs is contacting with the ground to support the robot body and propel the body to move forward. When the swinging set of legs finishes its SWP and starts its SSP, another set will finish its FSP and starts its SWP accordingly, and so on.

#### Variable rectilinear motion

Normally, when mobile robot receives the command of switching its locomotion pattern (e.g., changing the forward velocity), it is difficult for the trunk to reach the setting velocity immediately, because it requires all the actuators to accelerate or decelerate for a certain time. As a result, since the adjustment of rotational velocity of each actuator may take some time, so the real-time performance of robot may be compromised. On the other hand, if altering the rotational velocity of actuators within a wide range in a short time, it may damage the actuate components or transmission components. Therefore, for mobile robots, in consideration of safety of system, the adjustment time between two different locomotion patterns is necessary and inevitable.

Nevertheless, it is not true for ePaddle-based multi-legged robot. In the previous section, we have mentioned that the ePaddle-EGM is capable of altering the locomotion pattern smoothly and instantaneously. Since the *λ*_FST_ and *λ*_SSP_ could be arbitrary value (of course within its work space), when the duration of first/second stance phase is constant, the ePaddle-EGM can alter its forward velocity instantly, without sudden change in rotational velocity and reciprocating rotation of each driving joint. In other word, the switch between different locomotion patterns can be realized immediately. This non-reciprocating strategy can also be applied in the ePaddle-based hexapod robot to realize the fast transition of locomotion pattern. We will validate this in next section.

#### Turning motion

Turning gait is very important for the mobility of robot. Since each ePaddle-EGM module only has three DOFs in the plane of shell, the leg (supporting paddle) actuation is limited to motion in the sagittal plane. However, via differential motion between left and right modules, it is possible for ePaddle-based hexapod robot to realize turning motion. Due to unidirectional rotation of all the actuators during the legged gait, each ePaddle-EGM module cannot move in the opposite direction. Hence, the robot cannot turn in place. But through configuring different strides for legs of both sides, the robot can turn during running. In order to verify the ability to turn, the robot is simulated to turn with varying radii in this paper.

## Results and discussion

In this section, we conducted simulations to verify our proposed non-reciprocating legged walking gait for an ePaddle-based robot. The robot contains a trunk body and six ePaddle-EGM modules. For the simplicity, the configuration of the body is a rectangular parallelepiped. The simulations were conducted in multi-body dynamics simulator called Recurdyn (FunctionBay, Inc.). In these simulations, test trials are performed on an even terrain. The mass and inertia properties of the ePaddle component are extracted from CAD software. The coefficient of friction between the terrain and tips of paddle is taken to be equal to 0.8. The simulations have been performed with two cases: (1) variable rectilinear motion, which means walking along a straight-line path with different velocities, and (2) turning motion with varied radii. Since the uniform rectilinear motion is a relatively simple behavior, so the simulation of it has been omitted here.

**Fig. 9:**
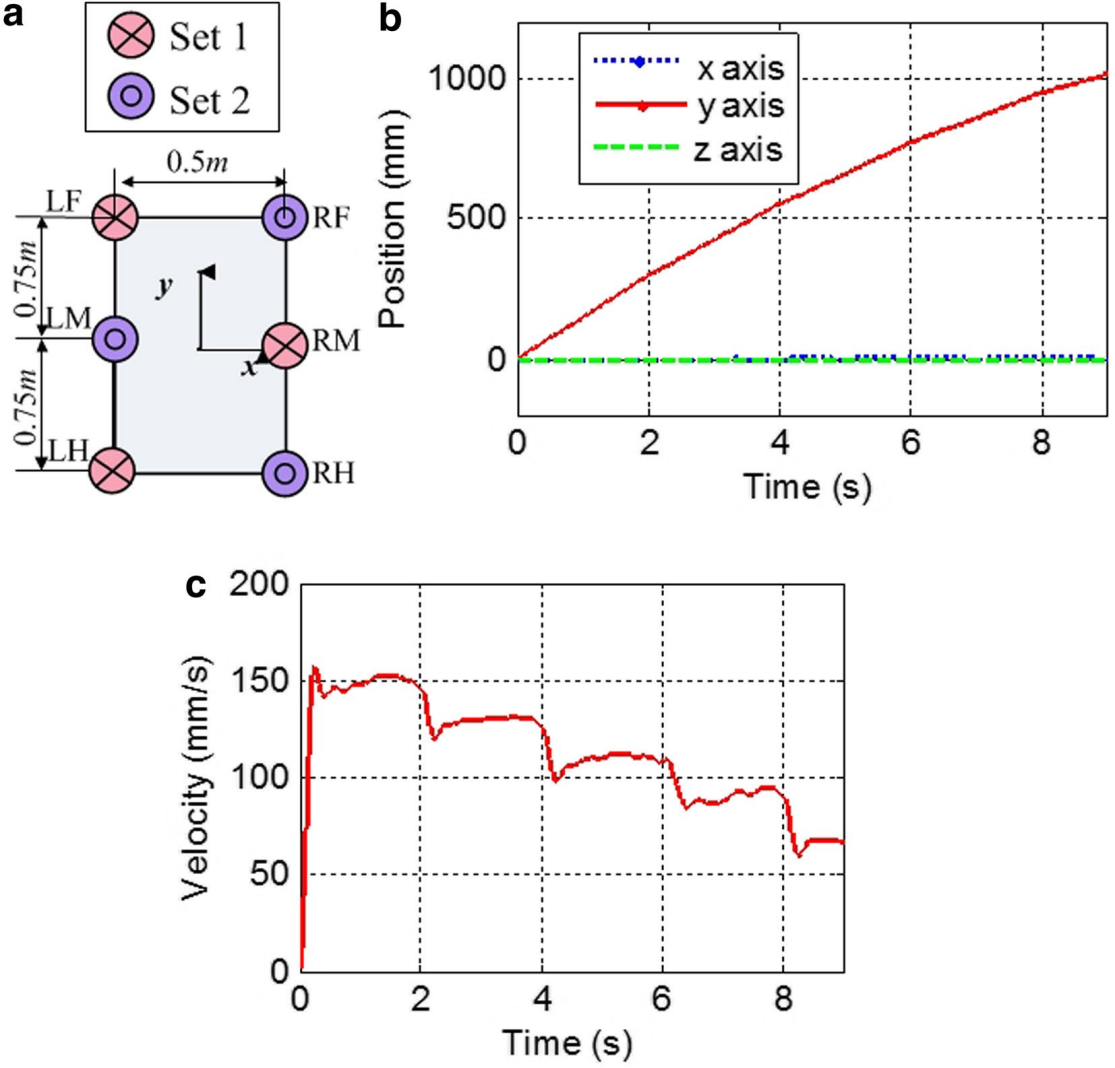
Simulated results for tracking a *straight-line* path with varied strides, while the cycle time *T* = 2 s. **a** Body size of robot. **b** Trajectory of the center of body. The *dotted line*, the *solid line* and the *dashline* signify the position on *x*, *y* and *z* axis, respectively. **c** *y*-axis velocity of the center of body

Figure 9 shows the simulation results for tracking a straight-line path with varied velocities. The body size of robot is shown in Fig. 6. Tripod gait is used in the simulation of hexapod robot. As what mentioned before, the six ePaddle-EGM modules are divided into two sets. Each set contains the front and rear modules of one side and middle module of the other side. Each module of the same set moves identically. Two sets of modules support the body alternately. In each ePaddle-EGM module, the duration of support phase and swing phase is the same. The static stability margin (SSM) proposed by McGhee and Frank [19] is employed as the stability criteria for the hexapod robot. The SSM is the shortest value of the distance from the projection of the center of gravity of the body to the boundaries of the support polygon. For our case, minimum magnitude of SSM is 29.1 when the robot is walking with the largest *λ* (340 mm). Hence, the legged walking gait of the hexapod robot is stable enough if *λ* is in the range of its largest value.

**Fig. 10:**
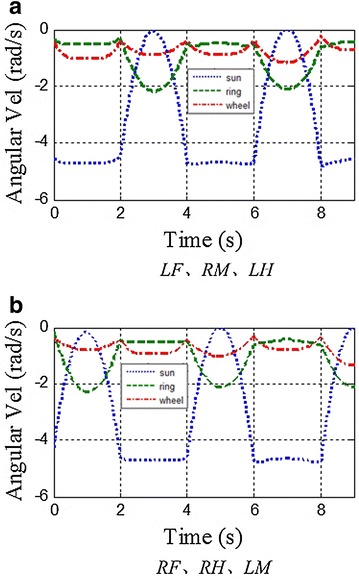
Angular velocities of actuators in the ePaddle-EGM when *λ* is 300–260–220–180–120 mm. **a** actuators in *LF*, *RH* and *LH*. **b** actuators in *RF*, *RH* and *LM*. The *dotted line*, the *dash line* and the *dot-and-dash line* signify the angular velocity of the sun gear, the ring gear and the wheel, respectively

Figure 9b shows the simulated results for tracking a straight-line path with varied strides. Simulated results confirm that our ePaddle-EGM-based robot can walk along a straight line with the proposed legged walking gait. Furthermore, *λ* of the gait during each gait period is different. The sequence of *λ* is 300–260–220–180–120 mm. Figure 13c shows the body’s velocity along *y*-axis, which indicates that there is a sudden change in the velocity of the body. However, Fig. 10 shows that all velocities of actuators are still continuous and unidirectional, which means the smooth switch of locomotion pattern can be realized through the proposed kinematic method.

**Fig. 11:**
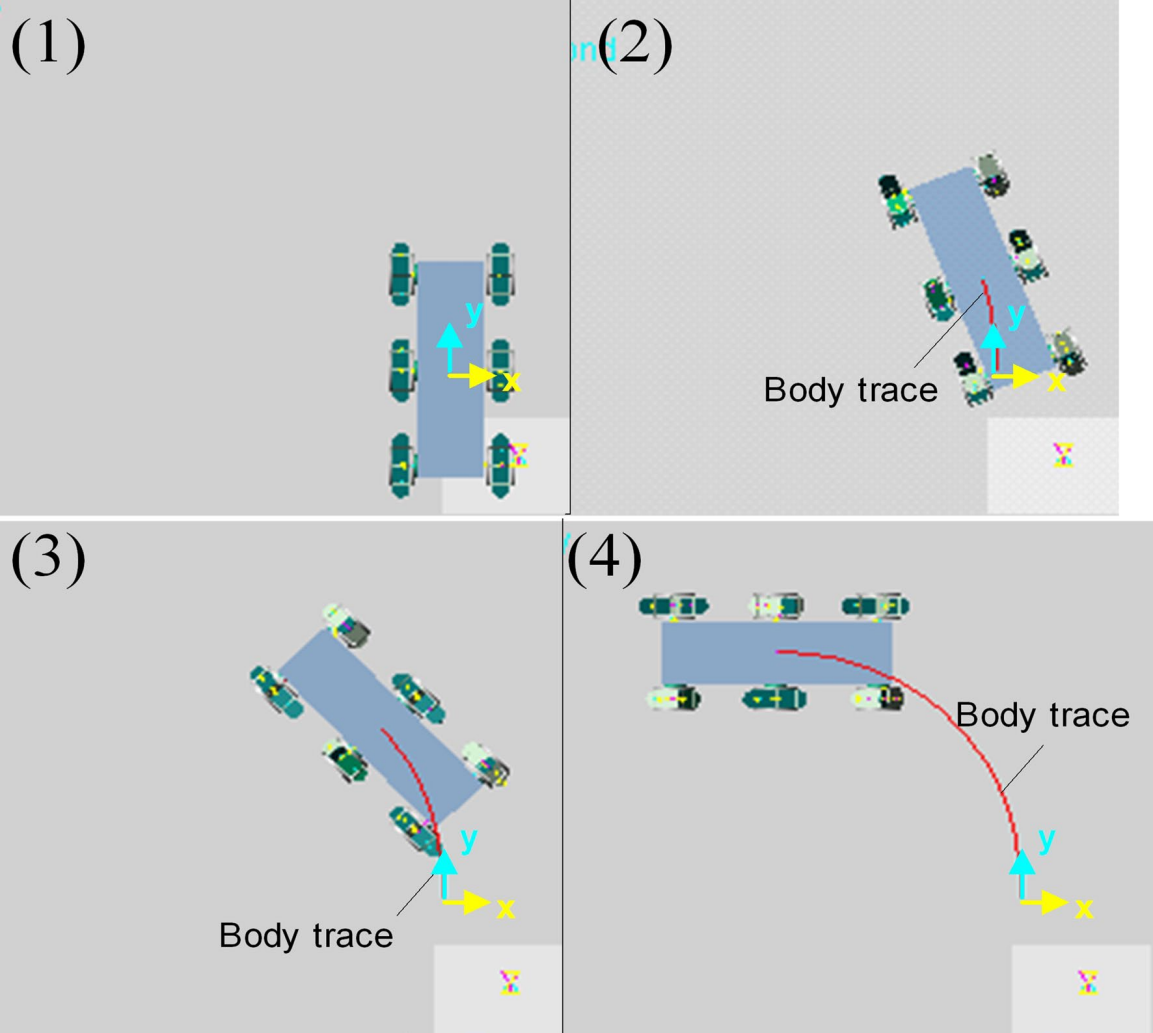
Snaps of the simulated result for the turning gait with the turning radius of 2 m. **1** initial pose. **2** and **3** snaps during turning. **4** end pose

**Fig. 12:**
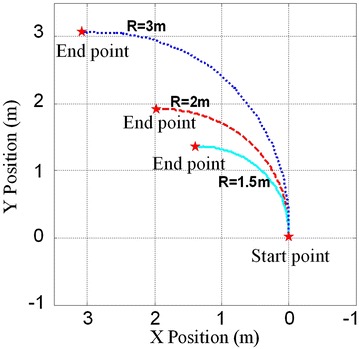
Simulated results of turning gait when the turning radii equals to 3 m (*dotted line*), 2 m (*dashline*) and 1.5 m (*solidline*)

**Fig. 13:**
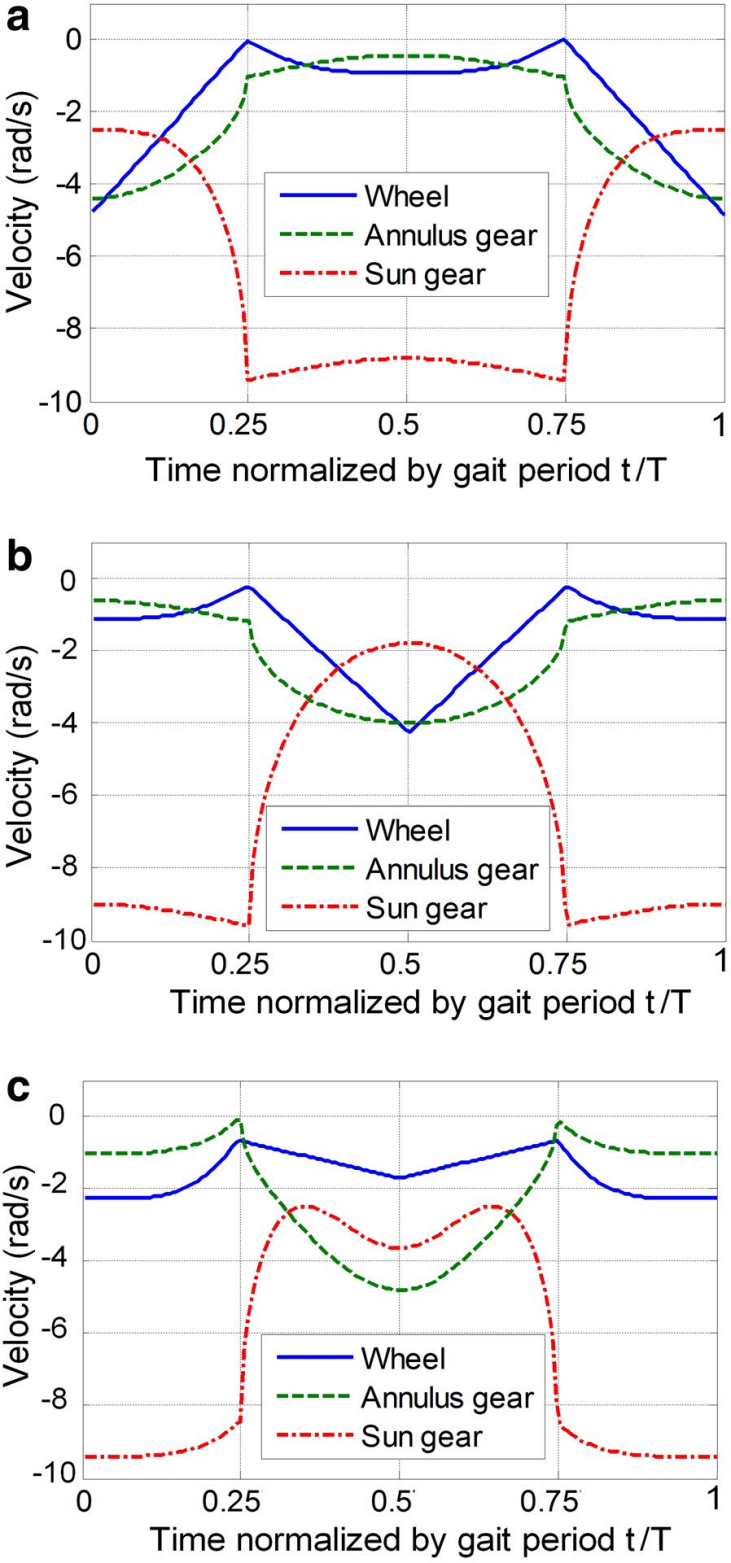
Angular velocities of the actuators for one gait cycle with the turning radius of 2 m. **a** Actuators in set 1. **b** Actuators in RF and RH. **c** Actuators in LM. The *solid line*, the *dashline* and the *dot-and-dash line* signify the angular velocity of the wheel, the ring gear and the sun gear, respectively

Turning gait is very important for the mobility of robot. Since each ePaddle-EGM module only has three DOFs in the plane of shell, the leg (supporting paddle) actuation is limited to motion in the sagittal plane. However, via differential motion between left and right modules, turning is possible. Due to unidirectional rotation of all the actuators during the legged gait, each ePaddle-EGM module cannot move in the opposite direction. Hence, the robot cannot turn in place. But through configuring different strides for both sides, the robot can turn during translation. In order to verify the ability to turn, the robot is simulated to turn with varying radii. Figure 11 shows the snaps of the simulated result for the turning gait. During turning, the modules in the same set are still synchronized internally. In these simulations, since turning to the left, the stride of modules in set 1 is zeros, which just support the robot when the modules in set 2 swing in the air. The stride of middle module on the left side (LM) is different from the stride of front and rear modules of right side (RF and RH). The turning radius varies with stride difference between LM and RF (RH). The simulated results are shown in Fig. 11. The three curves in Fig. 12 are the trajectories of the body when turning with different radii. In the three simulations, the stride difference is set to 320, 290 and 240 mm corresponding to the turning radius of 1.5, 2 and 3 m. We can conclude that the bigger the stride difference, the smaller the turning radius. When the difference is 0, the turning radius is infinite, which means walking along a straight line. Figure 13 shows the velocity of the actuators in one gait period when turning radius is 2 m. Figure 13 shows that the velocities of all the actuators are less than 0. Hence, all the actuators continually move in the same direction without reciprocating when robot is turning. This result verifies the mobility of the proposed legged walking gait.

## Conclusions

In this paper, a novel kinematic method for the ePaddle-EGM has been proposed. The distinguishing feature of this kinematic method is that it is capable of realizing discontinuous locomotion of the body through continuous and unidirectional rotation of actuators. In this way the influence of backlash in the epicyclic gear mechanism could be eliminated and accuracy of locomotion can be guaranteed. Based on this non-reciprocating kinematic method, we proposed the tripod gait planning method for the packsaddle-based hexapod robot to realize its stable motions, including the uniform rectilinear motion, the variable rectilinear motion and turning motion. The studies of simulations have been conducted to verify the performance of the tripod walking gait; namely, the hexapod robot can switch between different locomotion patterns instantly.
